# Analysis of the therapeutic gap for *Trypanosoma cruzi* infection in Colombia (2012 - 2022)

**DOI:** 10.1590/0074-02760260007

**Published:** 2026-10-16

**Authors:** Rafael Herazo, Ricardo Andrés Caicedo Díaz, Mauricio Javier Vera Soto, Andrea Liliana Sarmiento Ospina, Diogo Galvão, Andrea Marchiol, Maria-Jesus Pinazo

**Affiliations:** 1 Drugs for Neglected Diseases initiative, Rio de Janeiro, RJ, Brasil; 2 Ministerio de Salud y Protección Social, Dirección de Promoción y Prevención, Subdirección de Enfermedades Transmisibles, Grupo Gestión Integrada de Enfermedades Endemo Epidémicas, Colombia; 3 Universidad Autónoma de Manizales, Colombia

**Keywords:** access to treatment, health services accessibility, essential drugs, Chagas disease, Colombia

## Abstract

**BACKGROUND:**

In 2017, an estimated 1.2% of the population at risk for Chagas disease (CD) in Colombia was screened, and only 0.4% were treated. The recent implementation of CD-related Care roadmap has improved access to diagnosis and treatment. However, a therapeutic gap (TG) persists.

**OBJECTIVES:**

In this work, we aim to analyse the TG for *Trypanosoma cruzi*/CD infection in Colombia over the last years.

**METHODS:**

This is a descriptive study estimating the TG for *T. cruzi*/CD infection between 2012 and 2022. TG is defined as the number of people with a confirmed infection who have the indication of treatment but do not receive it. Two scenarios were considered in the TG analysis: (A) antiparasitic treatment is indicated in 70% of people with confirmed infection; (B) antiparasitic treatment indication in 85%. Data sources were official National and Departmental databases.

**FINDINGS:**

In scenario A, the TG was 77.5% in 2012, and by 2022, decreased to 48.1%. Two phases in the TG dynamics were observed: between 2012 and 2014, there was an increase of 4.5%; from 2014 onwards, there was a reduction of 40.7%. The most important reduction of TG was observed in 2018 and 2019. Linear projections from 2014-2022 indicate a potential reduction to 13.7% by 2030. Nevertheless, there are differences in terms of the reduction observed in different departments.

**MAIN CONCLUSION:**

TG calculation allows monitoring of treatment coverage, and its analysis facilitates target interventions to improve it, by reducing TG. Implementing interventions aimed at reducing the TG allows countries like Colombia to take a step forward in CD control.

Colombia is one of the 21 countries where Chagas disease (CD) is endemic. According to the World Health Organization (WHO), 4.8 million people in Colombia were estimated to be at risk of *Trypanosoma cruzi* infection in 2015.^1^ An epidemiological evaluation carried out in 2021 by the Ministry of Health and Social Protection (Minsalud) reported that up to 12 million people living in 596 of 1123 (53%) municipalities were at risk of infection by vector, congenital or oral transmission.^2^

Traditionally, CD has been viewed as a problem to be solved from the perspective of vector ecology, and preventive actions have been taken to control this type of transmission. Some progress has been made in Colombia through a better understanding of vector distribution.^3^ More importantly, during the last decade, progress has been made in interrupting intra-household vectorial transmission by *Rhodnius prolixus*, Colombia’s main vector, with 66 municipalities certified to date.^4^

In 2017, it was estimated that only 1.2% of the population at risk of CD in Colombia had been screened, and that only 68% of those who tested positive had access to confirmatory diagnostic testing, in accordance with the country’s official serological diagnostic algorithm. Only 0.4% of the people estimated to require trypanocidal treatment had access to it.^5^

In 2015, access barriers to timely care for people affected by CD in Colombia were documented. The identification of barriers and solutions was the basis for the Comprehensive Care Roadmap for Chagas disease (RIAS Chagas) developed by Minsalud with the support of DNDi in 2016.^6^

The diagnostic process was identified as a major barrier.^7^ In 2017, a new serological diagnostic algorithm for *T. cruzi* infection, based on enzyme-linked immunosorbent assay (ELISA) tests, was published by Colombia’s National Institute of Health.^8^ In the same year, pilot implementation of RIAS Chagas began, which was enacted by Minsalud.^6^

Between 2018 and 2019, a progressive implementation process began in the country to improve the detection and care of people with *T. cruzi* infection. However, a gap persists between diagnosed and treated patients.^9^ ^,^ ^10^

Therefore, the aim of this study was to estimate and analyse the therapeutic gap (TG) among individuals diagnosed with *T. cruzi* infection in Colombia between 2012 and 2022. Additionally, within the same timeframe, we sought to identify health promotion and prevention interventions, as well as care strategies targeting individuals affected by CD, and to describe their potential incidence on the dynamics of the TG.

## MATERIALS AND METHODS

A descriptive, cross-sectional study based on secondary sources was conducted to estimate the TG for *T. cruzi* infection/CD in Colombia between 2012 and 2022. The data required for the estimation were extracted from official secondary sources (see data source description below).

*Definitions* - There are several definitions for calculating gaps related to inequality indicators, including the arithmetic difference between observed data and reference data (poverty quintile, social groups, indicators, etc.).^11^ ^,^ ^12^ ^,^ ^13^ ^,^ ^14^

The Pan American Health Organization (PAHO) defines the treatment gap as follows: *“...the absolute difference between the number of people who present a condition and the number of people who receive appropriate care for that condition through health services. This can be expressed as the percentage of people who need treatment and do not receive it”.* ^15^ ^,^ ^16^

To calculate the gap using a direct method, it is necessary to conduct a prevalence study, as the number of people not receiving treatment is estimated based on the number of reported cases during the data collection process. On the other hand, the indirect method uses prevalence data and information on the amount of medication available in a given year.^16^

This study adopted the definition provided in the PAHO report and used an indirect method for the calculation. The TG for *T. cruzi* infection was estimated using the reported number of confirmed cases, the estimated proportion of people who met the criteria to initiate treatment regimen (two scenarios were considered, as defined below in the “GAP calculations assumptions” section), and individual prescriptions for trypanocidal treatment in Colombia.

*Study population* - The study was carried out using information obtained from official national data sources, with particular emphasis on the departments where RIAS Chagas had been implemented: Arauca, Boyacá, Casanare, Cundinamarca, Santander, Norte de Santander and Cesar.

*Gap calculation assumptions* - For *T. cruzi* infection/CD, we considered that trypanocidal treatment was not indicated in 100% of confirmed cases. According to current evidence-based guidelines, PAHO suggests not indicating trypanocidal treatment in adults with chronic infection and established organ-specific disease;^17^ such organ damage, mainly cardiac, is present in approximately 30% of people in the chronic phase.^18^ ^,^ ^19^ This was the first scenario used when calculating the TG (Scenario A). A second, alternative, scenario was also modelled, where therapeutic indications could be broader and more flexible, including people with functional class I and II heart disease (B1- B2 groups according to the AHA 2018 classification),^18^ increasing the therapeutic benefit to 85% of those infected (Scenario B). In addition, trypanocidal treatment was temporarily not indicated during pregnancy in women with chronic infection, as well as in the proportion of people reported in the literature as not wishing to receive trypanocidal treatment.^20^ Accordingly, we considered a theoretical scenario of 100 patients distributed according to clinical phase, the proportion eligible for treatment in chronic infection (scenarios A and B), the proportion of pregnant women,^21^ ^,^ ^22^ and the proportion of individuals who declined treatment. Based on these parameters, we calculated the proportion of patients who should initiate treatment; this proportion corresponds to the constant K used in the TG formula. In the case of Colombia, the constant K is practically the same as the proportion derived from the therapeutic indication scenarios for chronic infection. This is because the proportions of pregnant women and individuals declining treatment are very low and therefore have little impact on the calculation of the constant. Variations in these parameters — for example, an increase in the proportion of pregnant women with *T. cruzi* infection — would reduce the constant K (Table I). For Colombia, calculations were performed using the previously described values of 70% and 85% for scenarios A and B, respectively, and these values were also used as the K constant.

**TABLE I:** Parameters for estimating the therapeutic gap (TG)

	Scenario A		Scenario B	
Parameters^a^	p	n	p	n
Hypothetical number of cases		100		100
Proportion of acute cases^b^	0.07	7	0.07	7
Proportion of chronic cases^b^	0.93	93	0.93	93
Proportion of chronic patients with no indication for treatment^18^ ^,^ ^19^	0.3	28	0.15	14
Proportion of chronic patients with indication for treatment^18^ ^,^ ^19^	0.70	65	0.85	79
Proportion of patients who did not want treatment^20^	0.016	1	0.016	1
Proportion of pregnant women^c^ ^21^ ^,^ ^22^	0.05	0	0.05	1
*K* constant^d^	0.70	70	0.84	84

p: proportion values are expressed as decimals between 0 and 1; n: hypothetical number of cases

a parameters based on theoretical values;

b calculated based on a review of event reports from the National Institute of Health [2018-2019, 2021-2023 (first semester)]. See https://www.ins.gov.co/buscador-eventos/Paginas/Info-Evento.aspx.

c estimation based on the number of births among women of reproductive age;

d proportion of cases expected to initiate treatment: this includes acute and chronic cases eligible for treatment and excludes cases without treatment indication due to specific organ damage, pregnant women, and patients unwilling to initiate treatment.

*Sources of data* - Records derived from the National Public Health Surveillance System (SIVIGILA), the Health Service Information System (RIPS), and treatment prescription databases of the national and departmental vector-borne disease programs were reviewed as potential data sources for the number of cases and treatments provided.

The number of cases of CD in any clinical phase by department of residence reported between January 1, 2012 and December 31, 2022 was obtained from the annual reports of cases of CD published by the SIVIGILA, a source of public health data in Colombia.

Data on the number of individuals who initiated trypanocidal treatment were consolidated from the database of the National Chagas Subprogram of the Minsalud, which compiles data from the local vector-borne disease programs of the Departmental Health Secretariats of the 32 departments of Colombia.

Data from 2020 were excluded from the analyses because of the interruption in reporting on access to CD diagnosis and treatment during the severe acute respiratory syndrome coronavirus 2 (SARS-CoV-2) pandemic.

*Data quality and standardisation* - The data used in the present study were obtained from two information sources: the SIVIGILA and the treatment registry maintained by local vector-borne disease programs. Another available source was the RIPS, which were created to support the monitoring of reimbursement for health services within the system. However, after assessing their quality, consistency, and availability, these records were not included in the analysis.

To estimate the gap, data from official sources within the Colombian health system were used. Although these sources have inherent limitations related to the procedures employed during data collection, it is worth emphasising that the available data are nevertheless sufficient to support an initial analysis and exploration of the TG for this disease, particularly considering that this is the first such assessment conducted in the country.

In light of these limitations, we estimated the national gap using the cumulative totals for each variable. This means that it was not necessary to calculate an average gap across departments; instead, both the number of cases that initiated treatment and the number of confirmed cases of chronic *T. cruzi* infection were treated as absolute values rather than as results from subgroup analyses. We considered this result a national benchmark, thereby avoiding the heterogeneity that could have been introduced by a disaggregated analysis at the departmental or regional levels.

*Inputs for gap calculation* - Table I describes the parameters considered in the gap calculation.

Based on theoretical data, in a scenario without a TG, treatment would be initiated in 70.9% of individuals with *T. cruzi* infection (consistent with estimations of Scenario A).

*Statistical analysis: gap calculation* - The gap was calculated following two steps.

Step 1. The total number of cases during the study period (Nt) was multiplied by a constant (K) to estimate the number of cases with an indication for treatment (Ntx).

*Ntx* = *Nt* × *K*

Step 2. The TG is calculated with the following formula:^16^

*TG* = (*Ntx-N1*)/*Ntx* × 100

Where *N1* corresponds to the number of people who initiated treatment during the period.

The result of the calculation determines the size of the gap, expressed as a percentage.

The TG was estimated using a cumulative approach rather than separate annual analyses. This involved progressively adding the (N₁, Nₜ) values each year, using the cumulative totals for each annual calculation. This method allowed us to: (1) to standardise the annual variation in the TG, facilitating the identification of consistent patterns over the study period; (2) increase the accuracy of the estimates by taking into account that many patients started treatment years after diagnosis; (3) account for natural delays in treatment initiation, as treatment does not necessarily need to begin immediately according to clinical protocols; (4) assess the progressive impact of public health measures, whose effects usually become evident in the medium and long term.

*Data analysis* - The numbers of CD cases and patients initiating trypanocidal treatment per year were counted for the period 2012-2022, to identify the average annual notifications and the average percentage variation. Microsoft Excel 365 was used for the consolidation, cleaning and organisation of datasets, as well as for the generation of descriptive statistics and frequency distribution tables. The gap was estimated according to the methodology described above, and the percentage variation was estimated for various periods of interest. SPSS version 21 was used for advanced statistical analysis, with special emphasis on the estimation of relative epidemiological measures and their corresponding confidence intervals. Joinpoint Regression Program version 5.0.2 was used to implement segmented regression models, allowing the calculation of annual percentage change (APC) and the identification of significant inflection points in the analysed time trends. Finally, using Joinpoint model statistics, simulations of gap dynamics were performed using slope and intercept estimates in a simple linear regression model to project the behaviour of the gap for the period 2023 to 2030.

*Ethical considerations* - Official public records from Departmental and National information systems were used in the study. These secondary sources and data were aggregated and anonymised; no sensitive patient information was accessed or disclosed.

## RESULTS

Between 2012 and 2022, 4920 cases of *T. cruzi* infection were confirmed in Colombia, with an annual average of 492 (SD 163) cases. The annual number of notified cases ranged from 786 in 2012 to 260 in 2019. The number of notified cases of *T. cruzi* infection showed a downward trend, with an annual average decrease of 0.9%.

From 2012 to 2022, 1781 patients initiated antiparasitic treatment, with an average of 178 (SD 85.3) patients per year. The annual number of patients initiating treatment for *T. cruzi* infection ranged from 331 in 2019 to 71 in 2013. The trend in the initiation of trypanocidal treatment was upward, and it is estimated that on average it increased by 8.7% per year.

The estimated TG for *T. cruzi* infection in Colombia in Scenario A, for 2012 to 2022, showed a downward trend, with an estimated 38% reduction from 2012 (77.5%) to 2022 (48.1%). However, this decrease was not constant throughout the period. The analyses showed two trends in the dynamics of the gap: the first period was between 2012 and 2014, during which a 4.5% increase in TG was observed, from 77.5% in 2012 to 81.0% in 2014. From 2014 onwards, a 40.7% reduction in the gap was observed, from 81.0% in 2014 to 48.1% in 2022. The greatest annual reduction (15.3%) was observed between 2018 and 2019. The TG remained relatively stable from 2019 onwards (Fig. 1).

**Fig. 1:**
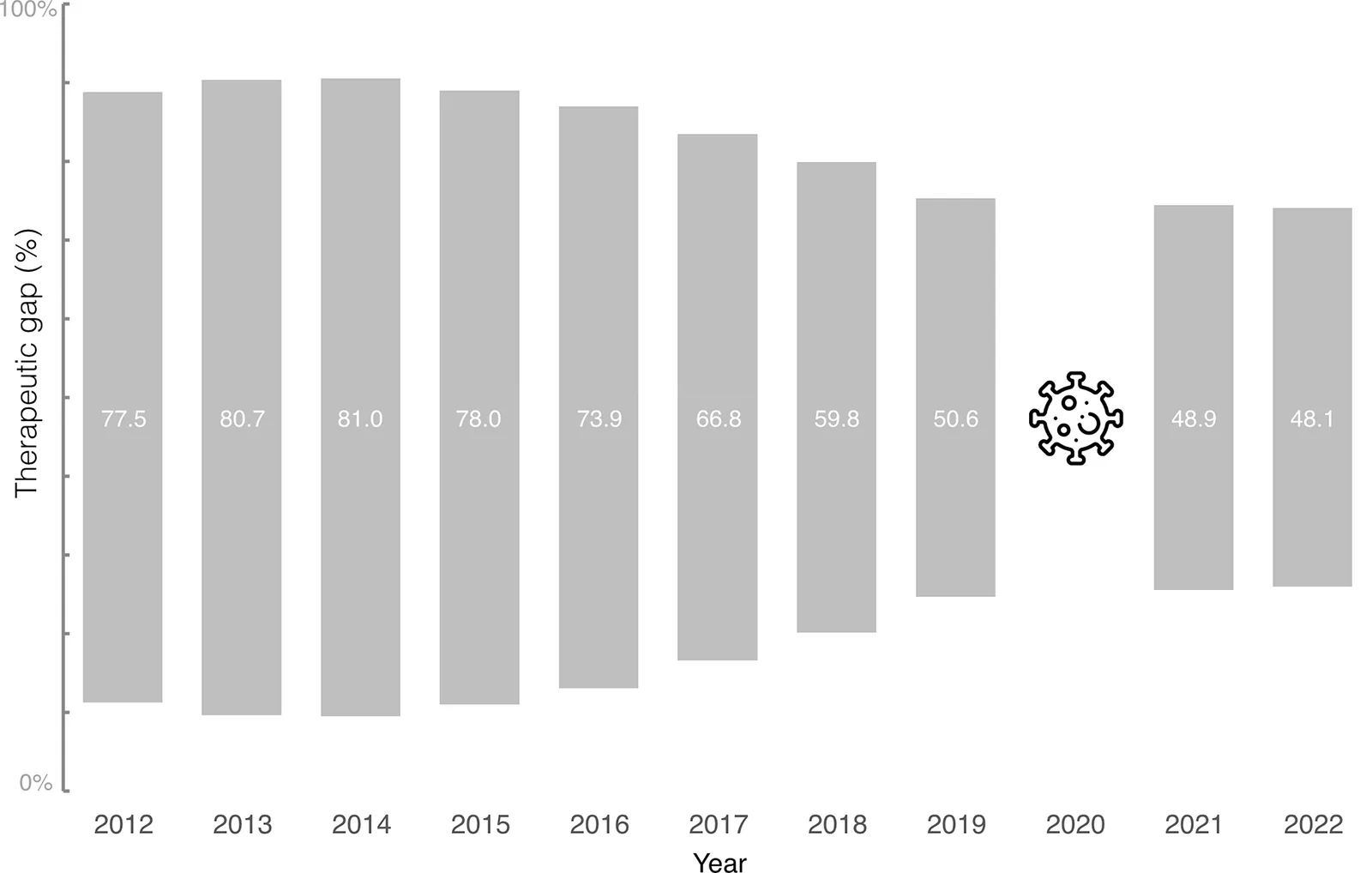
therapeutic gap (TG) for *Trypanosoma cruzi* infection in Colombia from 2012 to 2022 (Scenario 1). Note: No data were available for 2020 due to the impact of the Coronavirus disease 19 (COVID-19) pandemic on the health system and surveillance records.

These two trends were confirmed by a Joinpoint analysis, which showed that between 2012 and 2014 there was a slight increase in the gap, with an estimated APC of 2.72, and between 2014 and 2022 there was a decrease, with an APC of 7.17, with the trend in the second period being statistically significant (p < 0.05) (Fig. 2).

**Fig. 2:**
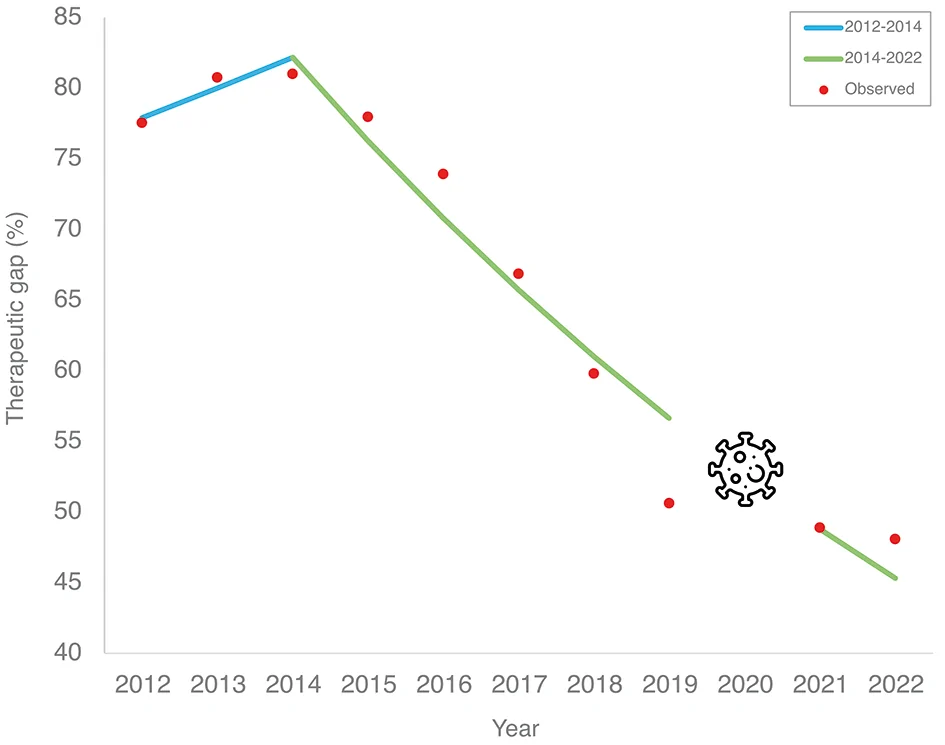
joinpoint analysis of the therapeutic gap (TG) for *Trypanosoma cruzi* infection, Colombia, 2012-2022. Note: The figure shows the lines emerging from the cumulative gap trend analysis, which connect to each other at the jointpoints. The blue line represents the trend of the first period between 2012 and 2014, and the green line represents the trend of the second period between 2014 and 2022. No data were available for 2020 due to the impact of the Coronavirus disease 19 (COVID-19) pandemic on the health system and surveillance records.

Estimates under scenario 2 clearly indicate a higher TG. Statistically, the trends observed during the periods 2012-2014 and 2014-2022 follow the same direction as in scenario 1 (Table II).

**TABLE II:** Calculation, trends and therapeutic gap (TG) projections by year and analysis scenarios

Variable	Scenario A	Scenario B
Year		
2012	77.5%	81.3%
2013	80.7%	83.9%
2014	81.0%	84.2%
2015	78.0%	81.6%
2016	73.9%	78.2%
2017	66.8%	72.3%
2018	59.8%	66.5%
2019	50.6%	58.8%
2021	48.9%	57.4%
2022	48.1%	56.7%
Trends and projections		
2012 - 2014	+ 4.5%	+ 3.5%
APC (p-value)	+ 2.72 (0.608)	+ 1.93 (0.655)
2014 - 2022	- 40.6%	- 32.6%
APC (p-value)	- 7.17 (< 0.000)	- 5.46 (< 0.000)
2012 - 2022	- 38%	- 30.3%
APC (p-value)	- 5.92 (< 0.000)	- 4.52 (< 0.000)
TG projection to 2030^*^	13.4%	27.7%
2012 - 2030	- 82.7%	- 66%
APC (p-value)	- 8.78 (< 0.000)	- 5.86 (< 0.000)

APC: annual percent change;

* Using linear regression with data from 2012-2022.

A department-level analysis of the TG for *T. cruzi* infection found that 28.1% (9 of 32) of departments in Colombia had the data necessary to estimate the TG. These departments exhibited heterogeneous TG patterns, including departments with a continuous decline over time (*e.g.*, Boyacá), in contrast to departments with no improvement in TG (*e.g.*, Arauca), and others in which an initial declining pattern was followed by an increase in TG (*e.g.*, Cesar). Visualising these patterns supports the use of TG estimation and analysis as a monitoring and evaluation tool (Fig. 3). In 59.4% (19) of the departments, no information related to the initiation of etiologic treatment in patients with CD was identified, and in 12.5% (4) of the departments the number of initiated treatments was significantly higher than the number of cases recorded. This situation may be explained primarily by four factors. First, during the period analysed, SIVIGILA did not require notification of chronic infections in non-Indigenous men older than 18 years; however, treatment may still have been provided and recorded in these cases. Second, some locally implemented strategies to reduce barriers to treatment access included dispensing medication without requiring completion of the SIVIGILA notification form. Third, internal migration within Colombia may also explain this discrepancy, as some patients may have been diagnosed in one department but initiated treatment in another. Finally, delays in treatment initiation may occur, which may lead to these processes being reported in consecutive years.

**Fig. 3:**
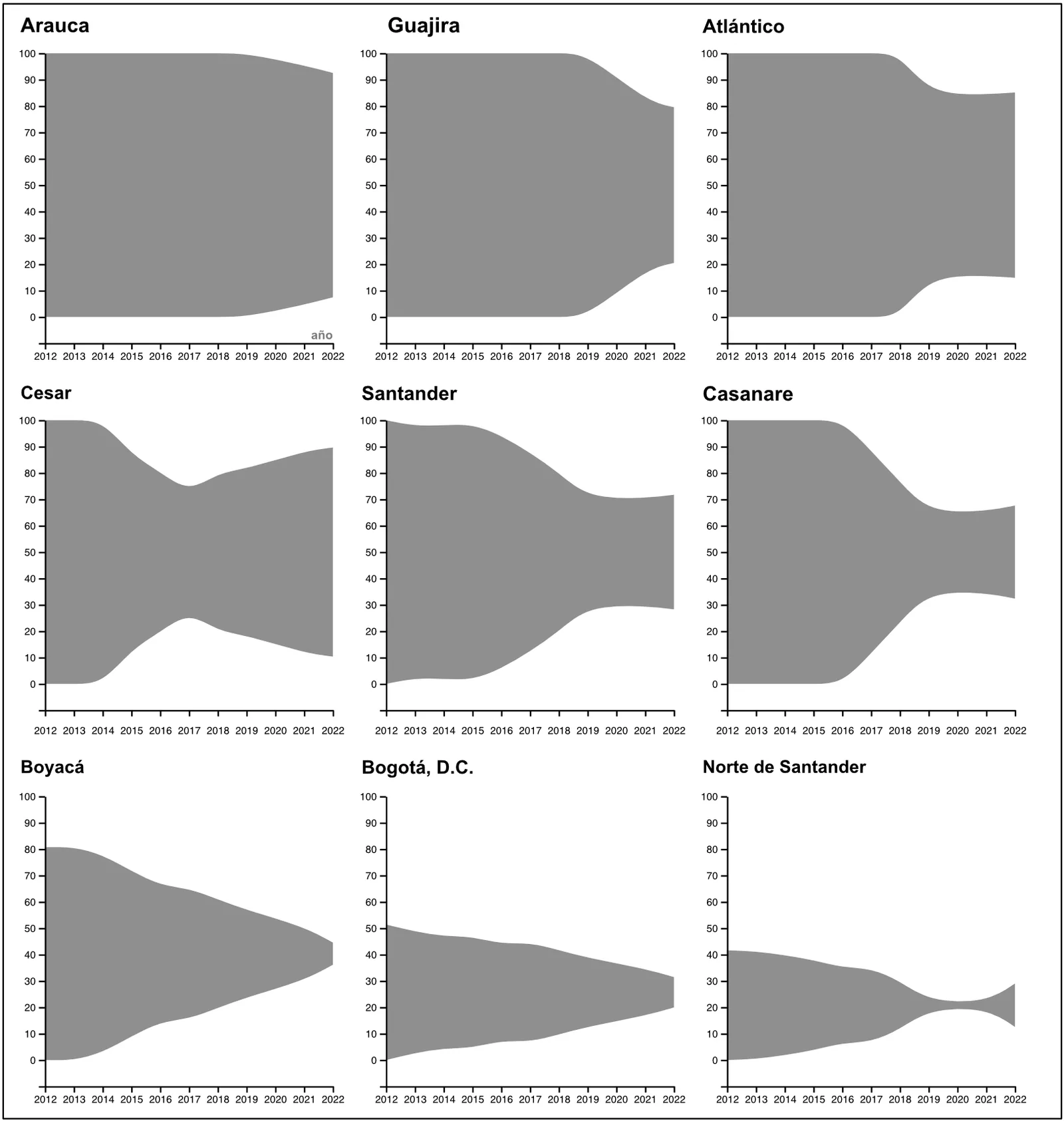
heterogeneity in Therapeutic Gap Patterns Across 9 Colombian Departments, 2012-2022.

The departmental analyses revealed that, as of 2019, the gap decreased in the endemic departments of Arauca and La Guajira. Arauca achieved a 15% reduction in two years, while La Guajira achieved a 40.9% reduction in the same period. At the end of the period analysed, the gaps were 85.1% and 59.1%, respectively.

The department of Atlántico reduced the gap between 2018 and 2019 by 31.3% and, as of 2019, the gap remained stable at around 70%. On the other hand, Cesar, one of the departments with the highest disease burden in Colombia over the last five years, reduced the gap by 55.2% between 2014 and 2017. However, between 2017 and 2022, the gap increased by 77%. The departments of Santander and Casanare achieved a significant reduction in the gap between 2016 and 2022; Santander reduced it by 55.3%, closing the period with a gap of 43.5%, and Casanare reduced it by 64.7%, closing the period with a gap of only 35.3%.

In the department of Boyacá, a sustained reduction in the TG for CD was observed. In 2012, the gap was estimated at 80.7%, but by 2022 the gap was only 8.4%, thanks to a sustained reduction of 89.6% during this period. Bogotá, the Capital District, achieved a sustained reduction in the gap of 77.6%, reaching 11.5% in 2022. Finally, there was a significant reduction in the department of Norte de Santander between 2012 and 2021, resulting in a 95.7% reduction in the gap. However, a 15-percentage point increase was estimated for 2022 compared to 2021.

Using the model derived from the Joinpoint analysis, two simulations were carried out to identify the possible evolution of the TG (Fig. 4). The first simulation was performed using data from the first period of the Joinpoint model (2012-2014), which showed an increase of 4.5% in the gap. Assuming linear behaviour, the gap would reach 100% in 2026, indicating a continued increase or persistence of a very high gap.

**Fig. 4:**
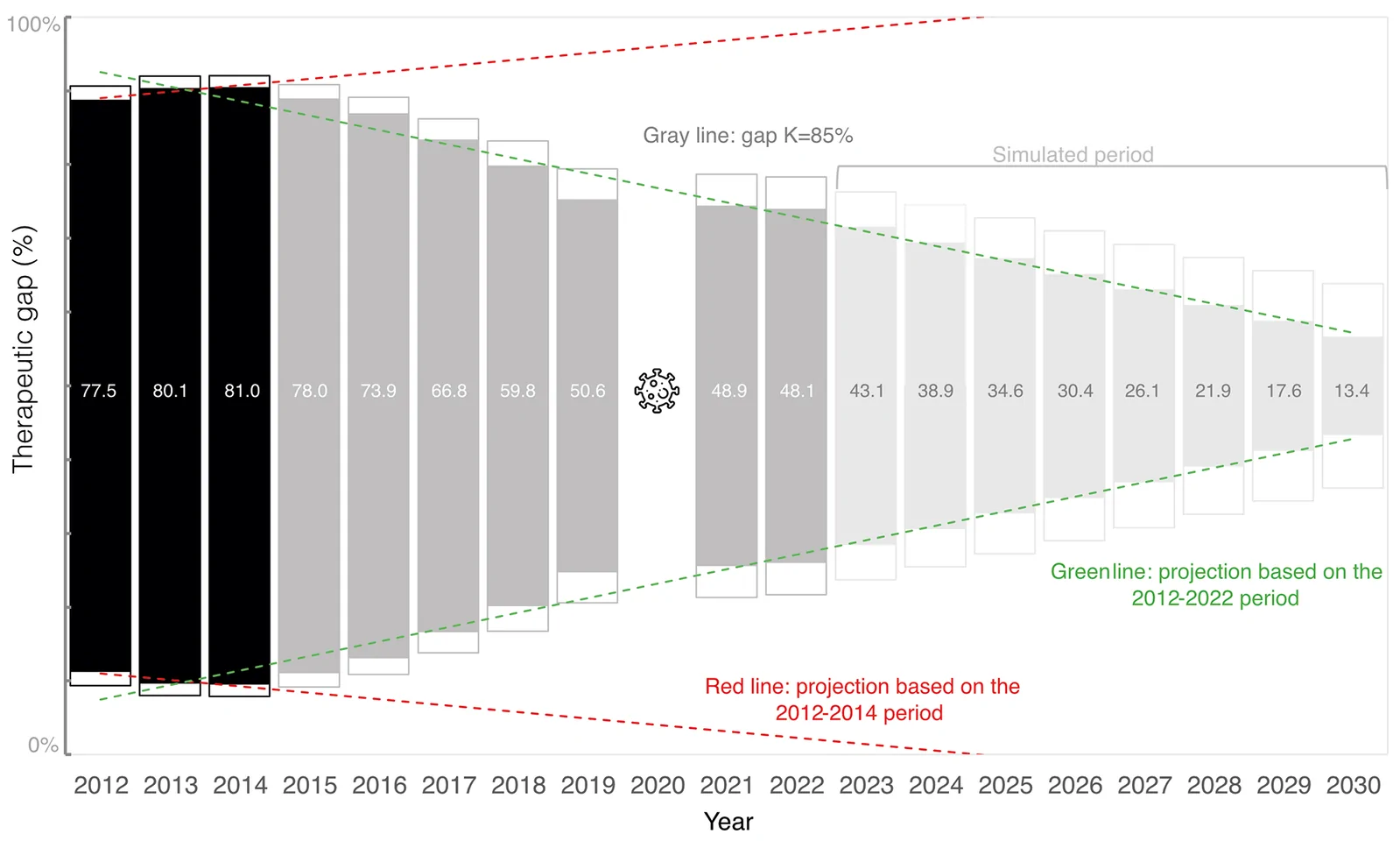
therapeutic gap (TG) dynamic simulations, Colombia, 2012-2030 - for scenarios A and B. Note: No data were available for 2020 due to the impact of the Coronavirus disease 19 (COVID-19) pandemic on the health system and surveillance records.

In the second simulation, the complete data set for 2012-2022 was used. In this period, a reduction of 38% was estimated. Assuming this trend remained linear, the gap would decrease to 13.4% by 2030, achieving a reduction of 82.7% over a period of 19 years.

## DISCUSSION

The TG is defined as the difference between the number of people who need medical care and the number of people who receive it, from identification to diagnosis and treatment.

TG analyses are widely used to assess deficiencies in health systems and to highlight their needs in terms of access for different pathologies of public health interest. They are frequently used to assess the evolution of chronic diseases and infectious and non-infectious conditions, including mental health events.^23^ ^,^ ^24^

TG is a public health impact indicator, as its reduction depends on a series of political and health care decisions that need to converge towards a common goal. These priority health policy decisions must work together to reduce the gap, which is closely related to the reduction of health inequalities.

Treatment gaps perpetuate an environment in which the right to health care is not guaranteed and reducing them is a major challenge and responsibility for health systems.

This is the first study to analyse the TG for *T. cruzi* infection/CD in Colombia, and it was conducted during a period in which: (1) a test and treat approach was adopted by the Chagas program in Colombia; and (2) a new diagnostic algorithm was implemented. Analysing the TG before and after these changes allowed national health authorities and other stakeholders to evaluate the impact of such actions. The analysis of TG showed a 38% decrease in the number of people left without treatment when it was deemed necessary during the ten years from 2012 to 2022.

In this study, we considered two scenarios. It is important to highlight that a substantial reduction was observed in both scenarios over the 10-year period evaluated. Scenario A assumed that treatment was prescribed for 70% of people with *T. cruzi* infection.^17^ This scenario was aligned with PAHO/WHO diagnostic and treatment guidelines and was based on the estimated proportion of people without severe organ involvement. In scenario B, the indication for treatment was expanded to include all people with *T. cruzi* infection without age-related contraindications, as well as patients with functional class I and II heart disease (2018 AHA group B1-B2 classification) (85%).

These findings place Colombia in a favourable position to advance toward the goal of eliminating CD as a public health problem.

In our study, we proposed two scenarios in which the fundamental difference lies in the expansion of the therapeutic indication to include cases with mild cardiomyopathy. In Colombia, transitioning from one scenario to the other would entail a set of challenges for the country.

The first challenge would be the time required to reduce the gap. Projections of the TG through 2030 indicate that, under Scenario B, the TG would be twice that of Scenario A. This suggests that more time would be required to achieve a greater reduction in the TG if current trends and the annual rate of reduction remain unchanged.

Additionally, an increase in the demand for medications would be expected, which would, in turn, require economic evaluation studies, particularly assessments of the budgetary impact on the healthcare system.

Under Scenario B, further requirements would include changes to clinical management guidelines, expanded continuing education to strengthen care delivery at both the primary healthcare level and in specialised medicine, as well as changes in regional policies regarding antiparasitic treatment.

When the TG is analysed by department, the results show that the trends and impacts are not uniform across departments. This behaviour could be due to local considerations, which were not part of the analysis for this study but are related to differences in the implementation of the Chagas care roadmap, as well as progress and sustainability in health promotion and disease prevention programs. The Coronavirus disease 2019 (COVID-19) pandemic substantially affected the health system in the country, as emergency care was prioritised, with negative consequences for overall healthcare coverage. Therefore, it was not possible to include data from 2020 because these data were unavailable in the surveillance system and other records. The post-pandemic recovery has also been uneven. Therefore, we acknowledge that data from 2021 and 2022 may still reflect a disrupted health system, and that trends estimated using data from 2019 to 2022 should be interpreted with caution.

In Colombia, two trends can be seen in the joinpoint regression analysis of the TG for CD. The first corresponds to the period between 2012 and 2014, during which there was a slight increase, although this period included only three years of data. The second corresponds to the period between 2014 and 2022, during which the gap showed a continuous and sustained reduction. This difference in trends may be explained by the greater efforts made between 2014 and 2022 to improve care for affected people, based on the implementation of chronic Chagas and maternal perinatal care roadmap in the country.^10^ This is consistent with the literature stating that efforts to eliminate CD as a public health problem must be comprehensive, encompassing promotional, preventive, and care actions.^25^

In the absence of intervention, the theoretical projection observed in Fig. 4, based on the estimations from 2012 to 2014, suggests that the TG would increase, worsening the clinical situation and treatment coverage in the context of Colombia.

A retrospective analysis suggests as a key starting point for progress toward disease control the Colombian Ministry of Health’s 2010 decision^26^ to certify the elimination of transmission by the main intradomiciliary vector tin approximately 100 prioritised endemic municipalities. This initiative, which is still in force today, built momentum for key subsequent decisions that were made to enable this reduction of the TG for CD in Colombia.

A seminar on the identification of access barriers related to CD in Colombia, held in 2015, was another important milestone. It triggered a series of policies and commitments by the Ministry of Health that focused on health care for people in the Colombian health insurance system.^7^

The resolution 3202 of 2016 for the implementation of the Comprehensive Healthcare Roadmaps (RIAS)^6^ was another important milestone. The CD RIAS incorporated a new and simplified ELISA-based algorithm from 2017, which allowed coverage to be extended to the entire country.^27^

The final major milestone within the period analysed was the implementation of the Maternal-Perinatal Care Roadmap in 2019. Based on the regional EMTCT-Plus initiative, it included systematic screening for CD and other prioritised diseases during pregnancy, using serological detection of IgG for *T. cruzi* infection.^9^

All initiatives have contributed to increased detection of cases and establishment of antiparasitic treatment, and this study showed that the treatment gap decreased substantially from 77.5% in 2012.

This study has focused on the analysis of records reported and documented in the different notification systems of the Ministry of Health of Colombia. Colombia does not have a harmonised and centralised system for the collection of specific data on *T. cruzi* infection/CD, especially for individual care and etiological treatment, and since universal notification of the chronic disease was not mandatory, it was therefore necessary to carefully analyse different sources of information, both at the national and departmental levels. Although an effort was made to rely on various sources, other information may have been unwittingly omitted from the analysis, potentially limiting the study.

Although this study does not include analysis of diagnostic coverage or diagnostic gap based on the population at risk, it is important to note that despite the country’s advances in access to Chagas diagnosis, with the implementation of a new algorithm in 2017,^8^ coverage still has limitations. A reduction in diagnosis trend in the first period was observed, potentially due to the non-uniform implementation of the new diagnostic algorithm and reporting systems across the country. Therefore, there is an unmet need for diagnosis that could influence the analysis of the TG.

While data for Colombia demonstrate a reduction in TG over time, what remains relevant from the patient’s perspective is that the gap persists, and although mathematical projections suggest a downward trend, the timeframe is long, and some people still do not access treatment. Therefore, the analysis of TG should include analysis of treatment access barriers as possible causes of TG persistence. This study did not include an analysis of the factors contributing to the TG or of patient-treatment seeking behaviour; however, it is known that in Colombia, the main barriers to treatment are related to, among other things, delays in distribution to municipalities, distance between rural patients and health centres, limited awareness of CD among physicians and patients, adverse drug reactions and lengthy treatment regimens.^7^

The lack of a comprehensive analysis of the heterogeneous dynamics of the TG across departments constitutes a limitation of this study. However, these dynamics may be attributable to the historical prioritisation of patient care activities, disparities in progress toward the implementation of the intradomiciliary vector transmission interruption plan, the adoption of a new ELISA-based diagnostic algorithm, and asymmetries in institutional responses to national commitments aimed at eliminating CD as a public health problem.

The calculation of the TG enables monitoring of treatment coverage, and its analysis facilitates the identification and prioritisation of targeted interventions aimed at reducing this gap. Implementing strategies to decrease the TG would allow countries such as Colombia to strengthen CD control by improving access to diagnosis and treatment, optimising resource allocation, and enhancing health system responsiveness. In this context, Colombia’s experience provides valuable lessons for other Latin American countries, highlighting the importance of integrating TG monitoring into national surveillance systems, aligning clinical guidelines with expanded treatment indications, and reinforcing capacity at both primary and specialised care levels. These lessons underscore the need for coordinated policy efforts, sustainable financing mechanisms, and continuous evaluation frameworks to effectively reduce the TG. Ultimately, addressing the TG represents a critical step toward mitigating disease progression, reducing morbidity, and advancing equitable public health outcomes across the region.

## Data Availability

After publication the data will be available on demand to corresponding author.
