# Supplementary material for: Analysis of the therapeutic gap for Trypanosoma cruzi infection in Colombia (2012 - 2022)
Source: Mem Inst Oswaldo Cruz. 2026 Oct 16;121:e260007. doi: 10.1590/0074-02760260007 (PMC13673982; doi:10.1590/0074-02760260007)
Supplement: Peer review history

# Reviewer report: FIRST REVIEW ROUND - REVIEWERS’ COMMENTS

## REVIEWER #1

Dear Authors,

This manuscript addresses a critically important public health issue regarding the persistent gap between diagnosis and treatment for Chagas disease in Colombia. However, some areas of the manuscript require clarification and improvement to enhance the rigor, clarity, and impact of the manuscript before publication.

Abstract

The abstract is well-structured, presenting key findings and the main conclusion. It successfully summarizes the central paper’s message. Although the finding that the percentage of therapeutic gap (TG) decreased from 77.5% to 48.1% was impactful, a projection to 13.7% by 2030, without the context of the assumptions behind this linear projection (as detailed later in the paper), can be misleading. The abstract should either state that this is based on a “linear projection of current trends” or omit the specific number and simply state that a continued reduction is projected. As a suggestion, in the “FINDINGS” section, rephrase the projection to: “Linear projections based on trends from 2014-2022 suggest a potential further reduction to 13.7% by 2030.” This adds the necessary methodological nuance.

Introduction

The introduction provides a concise background on Chagas disease in Colombia, covering topics ranging from vector control to the more recent focus on patient care. The logic flows well from problem identification to the study’s aim. However, the hypothesis was established rather briefly. Therefore, stating a more explicit research question would strengthen the paper. For example: “Has the implementation of the RIAS Chagas and the new diagnostic algorithm been associated with a measurable reduction in the therapeutic gap for T. cruzi infection in Colombia?” As a suggestion, conclude the introduction by preparing the study around a specific hypothesis or a set of research questions to guide the readers more directly to the methodology.

Methodology

The study design seems rather appropriate. However:

1. The description of the constant K and its calculation from Table 1 is confusing. It is unclear how the “Treatment goal” (0.709 in Table 1) is derived from the parameters above it. The manuscript states it is “based on theoretical data,” but there is no step-by-step demonstration of this calculation in the text or a supplementary file showing how each parameter in Table 1 contributes to the final value.

2. The article mentions data sources such as SIVIGILA and RIPS, but does not explain how discrepancies between these systems were managed. An important finding is that, in 4 departments, “...the number of initiated treatments was significantly higher than the number of cases recorded.” (page 12, lines 56 - 58). This critical issue of data quality is mentioned in the results, but its handling is not discussed in the methodology. This needs to be transparently addressed. As a suggestion, add a subsection of “Data Quality and Harmonization” to explicitly discuss how inconsistencies between data sources (e.g., treatments > cases) were identified and resolved, and what impact, if any, this had on the analysis.

3. Table 1 introduces “Pilot values” from reference 10, but the methodology does not explain how or why these values were used alongside “Theoretical values” in the analysis. Were they used to adjust the constant K for certain years or departments?

The authors should clarify the role of the “Pilot values” in Table 1. Were they used for sensitivity analysis? If so, describe that analysis. If not, remove them from the main table to avoid confusion.

Results

The results section presents a clear narrative of the overall national trend, identifying two distinct phases (2012-2014 increase, 2014-2022 decrease) with data supporting. However:

1. The results are presented almost exclusively for Scenario A. The reader is left wondering about the TG in Scenario B. If possible, the authors could include a figure or table summarizing the results for both Scenario A and Scenario B, even if only for the national level. This would provide a more complete picture. This is an opportunity to show the range of the gap and discuss the implications of different treatment guidelines.

2. The departmental results are descriptive but not analytical. Why did some departments succeed (Boyaca, Bogota) while others struggled (Cesar)? The article does not explore potential correlates of success, such as the timing of RIAS implementation, the capacity of the local health system, or departmental investment. As a suggestion, departmental analysis should go beyond simple description. In addition, the authors could create a table that, for each department, lists key variables (e.g., year RIAS implemented, number of cases, 2022 TG, % reduction) and briefly discusses potential factors that might explain the variation.

3. Although the reason for excluding 2020 is presented, the way it is presented (e.g., “break in trend” in the numbers) is acceptable. However, the impact of this missing year on the cumulative analysis should be addressed briefly. Does the gap post-2019 represent a true stabilization, or is it an artifact of the post-pandemic recovery that hadn’t yet been captured by 2022? The authors should add a sentence in the results or discussion acknowledging that the 2022 data may still reflect a disrupted healthcare system, and the trend from 2019 to 2022 should be interpreted with caution.

Discussion

The discussion provides a substantial narrative for the observed trends. Furthermore, it appropriately acknowledges the lack of a harmonized data system as a significant limitation.

The discussion mentions both scenarios (A and B) but does not delve into the policy implications of the different treatment indications. What would it take for Colombia to move towards the more inclusive Scenario B? This is a crucial point for the impact of this article. As a suggestion, expand the discussion to compare and contrast the implications of Scenarios A and B.

The text mentions barriers to treatment access in a single sentence (Page 21, lines 1-3). As a suggestion, the authors could dedicate a paragraph to a more detailed discussion of the persistent barriers, drawing not only on the cited article (Ref. 7), but also formulating hypotheses based on departmental results.

Conclusions

The conclusion is strong and directly answers the main objective. As a suggestion, the authors could include a summary of the main lessons learned for other countries or for the future of the program in Colombia.

Additional Minor Comments

Table 1: As cited in the methodology, inclusion of “Pilot Values” with “Theoretical Values” without explanation generates confusion. As a suggestion, remove the “Pilot Values” to a supplementary table and simplify Table 1 to show only the “Theoretical Values” used in the primary analysis. The footnote “d” should also be clarified.

Figures 1 and 2: These figures are well-organized, but a note regarding the 2020 pause would be helpful.

Figure 3: This figure is confusing and difficult to read. Perhaps a better approach would be to create a table showing the TG at key points in time (e.g., 2012, 2016, 2019, 2022) and the percentage change.

## AUTHORS’ RESPONSE TO THE REVIEWERS

We sincerely thank the reviewer for the careful evaluation of the manuscript and for the valuable comments provided. Below, we address each of the reviewer’s comments and provide a detailed response.

All final revisions and additions to the manuscript text are underlined in blue and can be seen in the newly uploaded version of the manuscript.

Reviewer’s comment – Abstract

The abstract is well-structured, presenting key findings and the main conclusion. It successfully summarizes the central paper’s message. Although the finding that the percentage of therapeutic gap (TG) decreased from 77.5% to 48.1% was impactful, a projection to 13.7% by 2030, without the context of the assumptions behind this linear projection (as detailed later in the paper), can be misleading. The abstract should either state that this is based on a “linear projection of current trends” or omit the specific number and simply state that a continued reduction is projected. As a suggestion, in the “FINDINGS” section, rephrase the projection to: “Linear projections based on trends from 2014-2022 suggest a potential further reduction to 13.7% by 2030.” This adds the necessary methodological nuance.

Authors’ Response – Abstract

Thank you for the thorough review.

We have accepted the suggestion and adjusted the text in accordance with the reviewer’s recommendation.

Text Included/Adjusted:

Linear projections from 2014–2022 indicate a potential reduction to 13.7% by 2030

Reviewer’s comments – Introduction

The introduction provides a concise background on Chagas disease in Colombia, covering topics ranging from vector control to the more recent focus on patient care. The logic flows well from problem identification to the study’s aim. However, the hypothesis was established rather briefly. Therefore, stating a more explicit research question would strengthen the paper. For example: “Has the implementation of the RIAS Chagas and the new diagnostic algorithm been associated with a measurable reduction in the therapeutic gap for T. cruzi infection in Colombia?” As a suggestion, conclude the introduction by preparing the study around a specific hypothesis or a set of research questions to guide the readers more directly to the methodology.

Authors’ response – Introduction

Thank you for the comments.

We have adjusted the text to improve clarity regarding the study’s objective and hypothesis. However, it was not the aim of this study to evaluate the impact of the implementation of the care roadmap or the change in the diagnostic algorithm, reaffirming the main objective of the study while incorporating the potential incidence of interventions throughout the analyzed period.

Text Included/Adjusted:

Therefore, the aim of this study was to estimate and analyze the therapeutic gap (TG) in those that were diagnosed for T. cruzi infection in Colombia between 2012 and 2022. Additionally, within the same timeframe, we sought to identify health promotion and prevention interventions, as well as care strategies targeting individuals affected by Chagas disease, and to describe their potential incidence on the dynamics of the therapeutic gap.

Reviewer comments – Methodology

The study design seems rather appropriate. However:

1. The description of the constant K and its calculation from Table 1 is confusing. It is unclear how the “Treatment goal” (0.709 in Table 1) is derived from the parameters above it. The manuscript states it is “based on theoretical data,” but there is no step-by-step demonstration of this calculation in the text or a supplementary file showing how each parameter in Table 1 contributes to the final value.

Authors’ response – Methodology

1. We revised the table 1 and added a new version to clarify the contribution of each parameter to the calculation of the gap for Colombia. In the manuscript text, we also included the conceptual definition of the constant K. See new table 1

Text Included/Adjusted:

Accordingly, we assumed and considered data from a theoretical scenario of 100 patients distributed by clinical phase, the proportion eligible for treatment in chronic infection (scenarios A and B), the proportion of pregnant women, and the proportion of individuals who declined treatment. Based on these parameters, we calculated the proportion of patients who should initiate treatment; this proportion constitutes the constant K applied in the TG formula. In the case of Colombia, the constant K is practically the same as the proportion derived from the therapeutic indication scenarios for chronic infection. This is because the parameters that would subtract cases (pregnant women and treatment refusal) are very low and therefore do not have a substantial impact on the calculation of the constant. Variations in these parameters—for example, an increase in the proportion of pregnant women with T. cruzi infection—would reduce the constant K. (see Table 1). For Colombia, calculations were performed using the previously described values of 70% and 85% for scenarios A and B, respectively, and these values were also used as the K constant.

Reviewer comments – Methodology

2. The article mentions data sources such as SIVIGILA and RIPS, but does not explain how discrepancies between these systems were managed. An important finding is that, in 4 departments, “...the number of initiated treatments was significantly higher than the number of cases recorded.” (page 12, lines 56 - 58). This critical issue of data quality is mentioned in the results, but its handling is not discussed in the methodology. This needs to be transparently addressed. As a suggestion, add a subsection of “Data Quality and Harmonization” to explicitly discuss how inconsistencies between data sources (e.g., treatments > cases) were identified and resolved, and what impact, if any, this had on the analysis.

Authors’ response – Methodology

2. The data included in this study were derived from two information sources: the Public Health Surveillance System (SIVIGILA) and the treatment registry of local vector-borne disease programs. An additional data source, the Individual Health Service Provision Records (RIPS), was also available. Although RIPS was originally developed to monitor reimbursement for health services within the Colombian health system, this source was ultimately excluded from the analysis after careful evaluation of its quality, consistency, and availability.

Following the reviewer’s recommendation, we included a subsection on data quality and standardization. In addition, three possible explanations were identified for the observation that, in four departments, the number of treated patients exceeded the number of diagnosed patients.

Text Included/Adjusted:

In the Methods section:

Data quality and standardization

The data used in the present study were obtained from two information sources: the Public Health Surveillance System (SIVIGILA) and the treatment registry maintained by local vector-borne disease programs. Another available source was the Individual Health Service Provision Records (RIPS), which were created to monitor reimbursement for health services within the system. However, after assessing their quality, consistency, and availability, these records were not included in the analysis.

To estimate the gap, data from official sources within the Colombian health system were used. Although these sources have inherent limitations related to the procedures employed during data collection, it is worth emphasizing that the available data are nevertheless sufficient to support an initial analysis and exploration of the diagnostic gap for this disease, particularly considering that this is the first such assessment conducted in the country.

In light of these limitations, we estimated the national gap using the cumulative total for each variable. This means that it was not necessary to average the gap across departments; instead, both the number of cases that initiated treatment and the number of confirmed cases of chronic T. cruzi infection were treated as absolute values rather than as outputs from subgroup analyses. We considered this result as a national benchmark, thereby avoiding the heterogeneity that could have been introduced by a disaggregated analysis at the departmental or regional level.

In the Results section

This situation may be explained primarily by four factors. First, during the period analyzed, SIVIGILA did not require notification of chronic infections in non-Indigenous men older than 18 years; however, treatment may still have been provided and recorded in these cases. Second, some locally implemented strategies to reduce barriers to treatment access included dispensing medication without requiring completion of the SIVIGILA notification form. Third, internal migration within Colombia should also be considered, as some patients may have been diagnosed in one department but initiated treatment in another. Finally, delays in treatment initiation may occur, which may lead to these processes being reported in consecutive years.

Reviewer comments – Methodology

3. Table 1 introduces “Pilot values” from reference 10, but the methodology does not explain how or why these values were used alongside “Theoretical values” in the analysis. Were they used to adjust the constant K for certain years or departments?

The authors should clarify the role of the “Pilot values” in Table 1. Were they used for sensitivity analysis? If so, describe that analysis. If not, remove them from the main table to avoid confusion.

Authors’ response – Methodology

3. The pilot values in Table 1 were included only as a reference to illustrate the difference with respect to the theoretical values. In line with the reviewer’s recommendation, and acknowledging that this column may cause confusion, we decided to remove it and provide a revised version of the table, as explained in comment 1 of the methodological comments.

Reviewer comments – Results

The results section presents a clear narrative of the overall national trend, identifying two distinct phases (2012-2014 increase, 2014-2022 decrease) with data supporting. However:

1. The results are presented almost exclusively for Scenario A. The reader is left wondering about the TG in Scenario B. If possible, the authors could include a figure or table summarizing the results for both Scenario A and Scenario B, even if only for the national level. This would provide a more complete picture. This is an opportunity to show the range of the gap and discuss the implications of different treatment guidelines.

Authors’ response – Results

1. For improved reader comprehension, we have included a descriptive table (see table 2) presenting the annual calculation of the therapeutic gap, along with its trends and projections under different scenarios. In addition, we have included a brief explanation in results section and discussion. Additionally, in the manuscript text, we corrected the percentage change in the therapeutic gap (TG) for the 2012–2022 period, as the value previously reported corresponded to the 2014–2022 period

Text Included/Adjusted:

In the results section

Estimates under scenario 2 clearly indicate a higher TG. Statistically, the trends observed during the periods 2012–2014 and 2014–2022 follow the same direction as in scenario 1. (See Table 2).

In the discussion section

In our study, we proposed two scenarios in which the fundamental difference lies in the expansion of the therapeutic indication to include cases with mild cardiomyopathy. In Colombia, transitioning from one scenario to the other would entail a set of challenges for the country.

The first challenge would be time. Projections of the therapeutic gap (TG) through 2030 indicate that, under Scenario B, the TG would be twice that of Scenario A. This suggests that more time would be required to achieve a greater reduction in the TG if current trends and the annual rate of reduction remain unchanged. Additionally, an increase in the demand for medications would be expected, which would, in turn, necessitate economic evaluation studies, particularly those prioritizing the budgetary impact on the healthcare system. Under Scenario B, further requirements would include modifications to clinical management guidelines, expanded continuing education to strengthen care delivery at both the primary healthcare level and in specialized medicine, as well as changes in regional policies regarding antiparasitic treatment.

Reviewer comments – Results

2. The departmental results are descriptive but not analytical. Why did some departments succeed (Boyaca, Bogota) while others struggled (Cesar)? The article does not explore potential correlates of success, such as the timing of RIAS implementation, the capacity of the local health system, or departmental investment. As a suggestion, departmental analysis should go beyond simple description. In addition, the authors could create a table that, for each department, lists key variables (e.g., year RIAS implemented, number of cases, 2022 TG, % reduction) and briefly discusses potential factors that might explain the variation.

Authors’ response – Results

2. The scope of the study is limited to describing the results without conducting analyses at the departmental level. We consider these suggestions important, and in the discussion section we will address several hypotheses that may potentially influence the differing dynamics of the therapeutic gap across departments.

We agree with this perspective. Please see the paragraph included in response to the reviewer’s suggestion under comment 2 in the section addressing the Discussion

Reviewer comments – Results

3. Although the reason for excluding 2020 is presented, the way it is presented (e.g., “break in trend” in the numbers) is acceptable. However, the impact of this missing year on the cumulative analysis should be addressed briefly. Does the gap post-2019 represent a true stabilization, or is it an artifact of the post-pandemic recovery that hadn’t yet been captured by 2022? The authors should add a sentence in the results or discussion acknowledging that the 2022 data may still reflect a disrupted healthcare system, and the trend from 2019 to 2022 should be interpreted with caution.

Authors’ response – Results

3. In line with the reviewer’s recommendation, we included in the Discussion section a paragraph addressing the possibility that the effects may have persisted into the post-pandemic period (2022), and that these data should therefore be interpreted with caution.

Text Included/Adjusted:

Therefore, we acknowledge that data from 2021 and 2022 may still reflect a disrupted health system, and that trends estimated using data from 2019 to 2022 should be interpreted with caution.

Reviewer comments – Discussion

The discussion provides a substantial narrative for the observed trends. Furthermore, it appropriately acknowledges the lack of a harmonized data system as a significant limitation.

1. The discussion mentions both scenarios (A and B) but does not delve into the policy implications of the different treatment indications. What would it take for Colombia to move towards the more inclusive Scenario B? This is a crucial point for the impact of this article. As a suggestion, expand the discussion to compare and contrast the implications of Scenarios A and B.

Authors’ response – Discussion

Thank you for the suggestion.

We have included the text below in the discussion:

Text Included/Adjusted:

1. In our study, we proposed two scenarios in which the fundamental difference lies in the expansion of the therapeutic indication to include cases with mild cardiomyopathy. In Colombia, transitioning from one scenario to the other would entail a set of challenges for the country.

The first challenge would be time. Projections of the therapeutic gap (TG) through 2030 indicate that, under Scenario B, the TG would be twice that of Scenario A. This suggests that more time would be required to achieve a greater reduction in the TG if current trends and the annual rate of reduction remain unchanged.

Additionally, an increase in the demand for medications would be expected, which would, in turn, necessitate economic evaluation studies, particularly those prioritizing the budgetary impact on the healthcare system.

Under Scenario B, further requirements would include modifications to clinical management guidelines, expanded continuing education to strengthen care delivery at both the primary healthcare level and in specialized medicine, as well as changes in regional policies regarding antiparasitic treatment.

Reviewer comments – Discussion

2. The text mentions barriers to treatment access in a single sentence (Page 21, lines 1-3). As a suggestion, the authors could dedicate a paragraph to a more detailed discussion of the persistent barriers, drawing not only on the cited article (Ref. 7), but also formulating hypotheses based on departmental results.

Authors’ response – Discussion

2. The barriers mentioned in the discussion section are those that persist over time, even after the country’s significant progress in providing

Text Included/Adjusted:

In the Discussion section

The lack of a comprehensive analysis of the heterogeneous dynamics of the therapeutic gap across departments constitutes a limitation of this study. However, these dynamics may be attributable to the historical prioritization of patient care activities, disparities in progress toward the implementation of the intradomiciliary vector transmission interruption plan, the adoption of a new ELISA-based diagnostic algorithm, and asymmetries in institutional responses to national commitments aimed at eliminating Chagas disease as a public health problem.

Reviewer comments – Conclusion

The conclusion is strong and directly answers the main objective. As a suggestion, the authors could include a summary of the main lessons learned for other countries or for the future of the program in Colombia

Authors’ response – Conclusion

Thank you for the suggestion. We have adjusted the last paragraph take into account the suggestion.

Text Included/Adjusted:

The calculation of the therapeutic gap (TG) enables monitoring of treatment coverage, and its analysis facilitates the identification and prioritization of targeted interventions aimed at reducing this gap. Implementing strategies to decrease the TG would allow countries such as Colombia to strengthen Chagas disease control by improving access to diagnosis and treatment, optimizing resource allocation, and enhancing health system responsiveness. In this context, Colombia’s experience provides valuable lessons for other Latin American countries, highlighting the importance of integrating TG monitoring into national surveillance systems, aligning clinical guidelines with expanded treatment indications, and reinforcing capacity at both primary and specialized care levels. These lessons underscore the need for coordinated policy efforts, sustainable financing mechanisms, and continuous evaluation frameworks to effectively reduce the TG. Ultimately, addressing the therapeutic gap represents a critical step toward mitigating disease progression, reducing morbidity, and advancing equitable public health outcomes across the region.

Additional Minor Comments

Reviewer comments

1. Table 1: As cited in the methodology, inclusion of “Pilot Values” with “Theoretical Values” without explanation generates confusion. As a suggestion, remove the “Pilot Values” to a supplementary table and simplify Table 1 to show only the “Theoretical Values” used in the primary analysis. The footnote “d” should also be clarified.

Authors’ response

Thank you for your feedback.

1. We revised the table 1 and added a new version to clarify the contribution of each parameter to the calculation of the gap for Colombia

Reviewer comments

2. Figures 1 and 2: These figures are well-organized, but a note regarding the 2020 pause would be helpful

Authors’ response

2. We added a brief statement to the footnotes of Figures 1 and 2 to clarify the absence of data for 2020.

Text Included/Adjusted:

Note: No data were available for 2020 due to the impact of the COVID-19 pandemic on the health system and surveillance records.

Reviewer comments

3. Figure 3: This figure is confusing and difficult to read. Perhaps a better approach would be to create a table showing the TG at key points in time (e.g., 2012, 2016, 2019, 2022) and the percentage change.

Authors’ response

3. Thank you for this valuable suggestion. We would like to clarify that Figure 3 illustrates the pattern of the therapeutic gap across 9 departments. Its purpose is to demonstrate the heterogeneity of the therapeutic gap at the local level, rather than to provide detailed individual analyses for each department. With this in mind, we would like to propose retaining Figure 3, while improving the image resolution and clarifying its purpose both in the figure title and in the corresponding section of the manuscript text.

Text of the adjusted title:

Heterogeneity in Therapeutic Gap Patterns Across 9 Colombian Departments, 2012–2022

Reference Text of Figure 3 in the text of the manuscript (Results):

These departments exhibited heterogeneous TG patterns, including departments with a continuous decline over time (e.g., Boyacá), in contrast to departments with no improvement in TG (e.g., Arauca), and others in which an initial declining pattern was followed by an increase in TG (e.g., Cesar). Visualizing these patterns supports the use of TG estimation and analysis as a monitoring and evaluation tool.

# Reviewer report: REVIEWERS’ COMMENTS

## REVIEWER #1

The authors have done a respectable job addressing the major concerns raised in the previous review. The revised manuscript shows substantial improvement in methodological transparency, data quality reporting, and the overall presentation of results. The core findings remain impactful, and the manuscript is now significantly closer to the standards expected for good publication. I congratulate the authors on their careful and professional revision.
